# Daily Self-Monitoring of Symptoms and Skills Learning in Patients With Borderline Personality Disorder Through a Mobile Phone App: Protocol for a Pragmatic Randomized Controlled Trial

**DOI:** 10.2196/17737

**Published:** 2020-05-25

**Authors:** Stig Helweg-Jørgensen, Mia Beck Lichtenstein, Alan E Fruzzetti, Christian Møller Dahl, Susanne S Pedersen

**Affiliations:** 1 Research Unit for Telepsychiatry and E-mental Health Mental Health Services in the Region of Southern Denmark Odense Denmark; 2 Institute of Psychology University of Southern Denmark Odense Denmark; 3 The Borderline Unit Mental Health Services in the Region of Southern Denmark Svendborg Denmark; 4 Odense Patient Data Explorative Network Odense University Hospital Odense Denmark; 5 Department of Clinical Research University of Southern Denmark Odense Denmark; 6 Department of Psychiatry Harvard Medical School Boston, MA United States; 7 Department of Business and Economics University of Southern Denmark Odense Denmark

**Keywords:** borderline personality disorder, dialectical behavior therapy, mobile app, psychotherapy, patient-reported outcome measures, mhealth

## Abstract

**Background:**

Patient self-monitoring via mobile phones during psychotherapy can enhance and provide an overview of psychotherapeutic progress by graphically displaying current and previous symptom scores, providing feedback to the patient, delivering psychoeducative material, and providing timely data to the therapist or treatment team.

**Objective:**

This study will aim to assess the effects of using a mobile phone to self-monitor symptoms and acquire coping skills instead of using pen and paper during psychotherapy in patients with borderline personality disorder (BPD). Dialectical behavior therapy will be performed to treat BPD. The primary outcome is the mean time needed to learn coping skills directed at emotion regulation; the secondary outcome is changes in the BPD symptom score as measured by the Zanarini Rating Scale for Borderline Personality Disorder.

**Methods:**

This study is a pragmatic, multicenter randomized controlled trial. Participants were recruited through five public general psychiatric outpatient treatment facilities in Denmark. Patients are randomly assigned, on a 1:1 basis, to either the mobile phone condition (using the Monsenso mDiary mobile app) or pen-and-paper condition. Patients will complete several self-report questionnaires on symptom severity; assessments by trained raters on BPD severity will be performed as well. Survival analysis with a shared frailty model will be used to assess the primary outcome.

**Results:**

Recruitment began in June 2017 and was completed in February 2019 after 80 participants were recruited. The study ended in February 2020. It is expected that the benefits of mobile phone–based self-report compared to the pen-and-paper method will be demonstrated for skill learning speed and registration compliance. To our knowledge, this is the first trial exploring the impact of cloud-based mobile registration in BPD treatment.

**Conclusions:**

This trial will report on the effectiveness of mobile phone–based self-monitoring during psychiatric treatment. It has the potential to contribute to evidence-based clinical practice since apps are already in use clinically.

**Trial Registration:**

ClinicalTrials.gov NCT03191565; https://clinicaltrials.gov/ct2/show/NCT03191565

**International Registered Report Identifier (IRRID):**

DERR1-10.2196/17737

## Introduction

The prevalence of borderline personality disorder (BPD) in the general Scandinavian population is estimated to be 1% to 5% [1,2]. The consensus is that approximately 1.5% of the western population meets the criteria for BPD [2]. The prevalence in clinical populations is considerably higher and is estimated to be around 28%, ranging between 9.3%-46.3% of patients, according to current studies [3,4]. In Scandinavia, the mortality risk of patients with a mental disorder is 2-3 times higher than in the general population [5]. The suicide rate for BPD patients is estimated to be between 8%-10%, almost 50 times higher than in the general population [6].

BPD is characterized by instability in emotion and mood, interpersonal relationships, self-image and identity, and impulse and behavioral control [7]. A 3-factor structure has been found empirically and supported by confirmatory factor analysis; the factors were disturbed relatedness, behavioral dysregulation, and affective dysregulation [8]. In dialectical behavioral therapy (DBT), these problems are viewed as skill deficits that result from problems with regulating emotion [9].

DBT has demonstrated effectiveness and is regarded as one of the most well-researched, evidence-based treatments for BPD [10-12]. The treatment includes the “five functions” of DBT—skill acquisition, skill generalization, motivation to implement new and skillful behaviors, interventions in the social and family environment to allow for treatment progress, and a consultation team to facilitate skillful treatment delivery and reduced burnout among therapists. Thus, the central focus is on learning skills that target self-management through mindfulness skills, healthier relationships with family and peers through interpersonal skills, handling of severe emotional dysregulation through distress tolerance and crisis survival skills, and proactive, effective management of emotional reactions through emotion regulation skills [13]. All of these types of skills are typically trained in a group format, and motivation and implementation are the foci of individual therapy.

### Self-Monitoring During Therapy

Self-monitoring of skill use and accompanying changes in suicidality, self-harm, and emotional reactivity during DBT therapy have traditionally been done using paper-based diaries. Technological advances in mobile apps have made new modes of self-monitoring possible and may reduce the burden on patients, increase data quality, and generate new opportunities for registration [14,15], like enhanced overview [16], ecological momentary assessment [17], and research investigating predictors of the course of therapy to facilitate future development [18,19]. However, monitoring of patients with BPD on mobile phone diary apps should be explored and evaluated before they are implemented in clinical practice in a broader sense [20].

Recent studies on pain management have demonstrated good usability in using digital self-monitoring [21,22]. Furthermore, studies using digital diaries in the treatment of bipolar disorder [19,23] in pain and weight management, sleep, and chemotherapy have all shown promising results [24-27]. Apps specifically targeting emotional awareness, posttraumatic stress reduction, and suicidality in borderline personality disorder are currently being investigated [28-30]. DBT skills have been shown to mediate improvements in BPD defining behaviors [31-35]. DBT-related apps supported by scientific inquiry have been developed at Rutgers University (Pocket Skills) [36] and the University of Washington (DBT Coach) [37]. These apps have been reported to show promise and acceptability among users. They are specialized in training and coaching skills, include diary card data as a secondary feature, and are self-contained within the app. The end users in the Pocket Skills usability study requested enhanced visualization of diary card scores as well as aggregated scores.

In this study, we used the mDiary app to fill this gap in research as well as to eliminate conventional paper diary cards through new technology. The mDiary app has a cloud-based self-monitoring system that is sharable with a therapist in real time. To our knowledge, this app is the first BPD-focused mobile app to provide sharable self-monitoring.

The Monsenso system used in our study is a modified version of the system used in the 2009 MONARCA trial, which tested a system aimed at self-monitoring bipolar disorder [38]. The MONARCA-project was developed at the IT University of Copenhagen as part of a PhD project, and a modified version of the MONARCA software is now sold by the Danish company Monsenso. The Svendborg DBT Unit modified the Monsenso mobile app to suit the needs of patients with BPD; modifications were made to the DBT treatment skills-training modules and psychoeducation content, and an enhanced therapist overview screen was added to the Monsenso system. Patients were involved in the design of the solution. A consistent focus on emotion regulation, the monitoring of progress in skills training, and compliance with standard DBT treatment [39] were prioritized in the mDiary app. The resulting solution was tested in a pilot feasibility study and showed adequate usability among patients and therapists [40].

### Objectives

The objectives of the current study are to evaluate (a) if patients randomized to use the Monsenso mobile app learn DBT skills faster compared to patients randomized to the pen-and-paper version; (b) if patients using the app report higher reductions in BPD criteria; (c) if registration compliance improves with a mobile phone, and (d) if use of the mobile app is cost-effective compared to the pen-and-paper version. We expect that use of a mobile phone–based digital diary will reduce the time it takes for patients to acquire DBT skills, improve therapy outcomes, and be cost-effective.

## Methods

### Study Design

**Figure 1:**
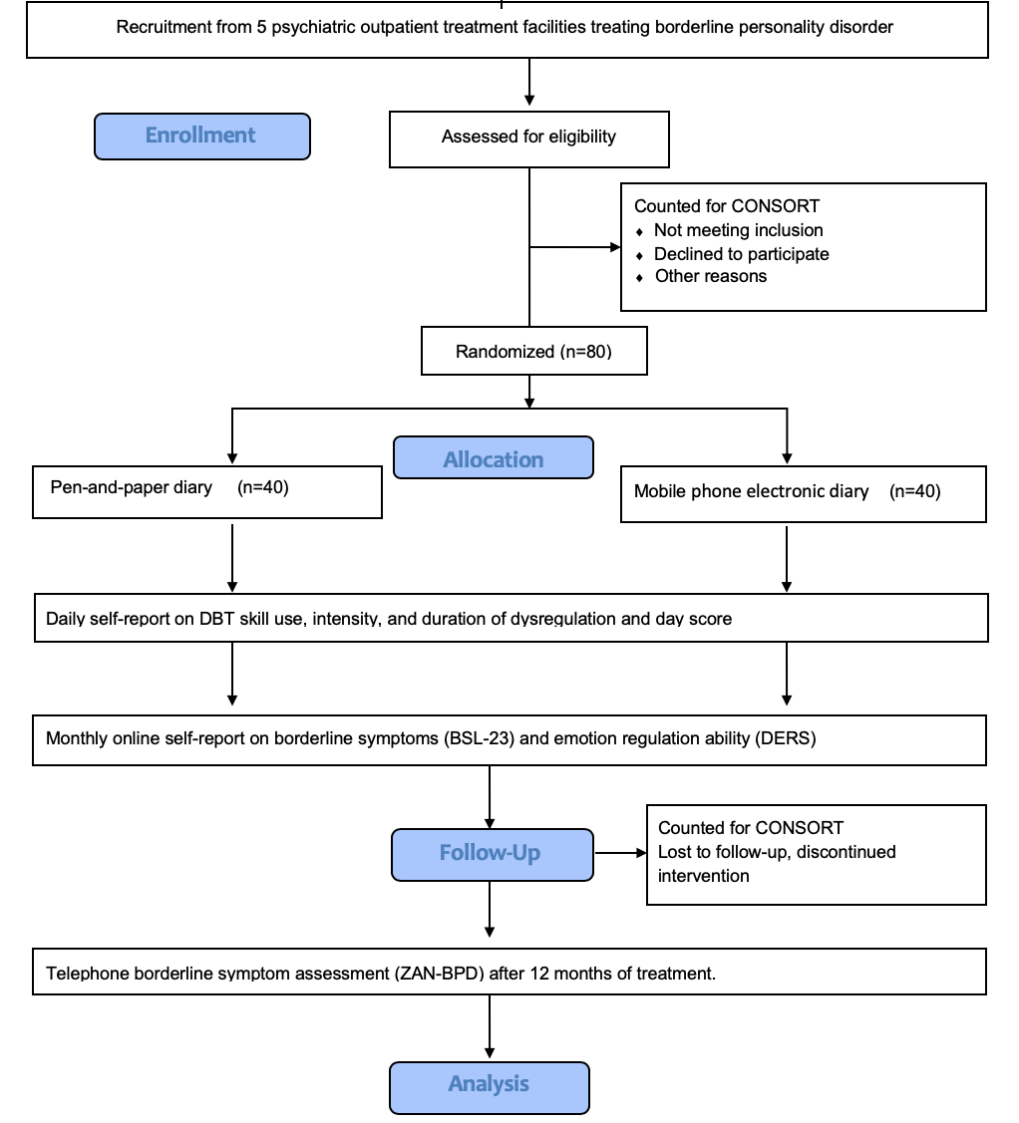
CONSORT (Consolidated Standards of Reporting Trials) flowchart. DBT: dialectical behavioral therapy; DERS: Difficulties in Emotion Regulation Scale; BSL-23: Borderline Symptom List; ZAN-BPD: Zanarini Rating Scale for Borderline Personality Disorder.

The study is a pragmatic 2-arm, multicenter, open-label, evaluator-blind, superiority randomized controlled trial (RCT), with the active arm being self-registration done through the Monsenso mDiary mobile app and the control arm being self-report done by pen-and-paper diary cards. Figure 1 presents the CONSORT (Consolidated Standards of Reporting Trials) flowchart and details on patient inclusion, and Table 1 provides an overview of the study.

**Table 1:** Items from the trial registration data set.

Data category	Information
Primary registry and trial identifier	ClinicalTrials.gov (NCT03191565)
Date of registration in primary registry	June 19, 2017
Secondary identification numbers	S-20160085 5159-00002B 2008-58-0035
Source of monetary or material support and sponsor	The Danish National Innovation Fund (grant number 5159-00002B)
Contact for public queries	Centre for Telepsychiatry, Odense, Denmark
Contact for scientific queries	Research Unit for Telepsychiatry and E-mental Health, Odense, Denmark
Public title	Differences in electronic and paper-based self-monitoring in borderline personality disorder: which is most effective?
Scientific title	Daily Self-Monitoring of Symptoms and Skills Learning in Patients With Borderline Personality Disorder Through a Mobile Phone App: Protocol for a Pragmatic Randomized Controlled Trial
Countries of recruitment	Denmark
Health condition(s) or problem(s) studied	Borderline personality disorder
Intervention(s)	Active comparator: self-monitoring by mobile phone Placebo comparator: *self-monitoring by pen and paper*
Key inclusion and exclusion criteria	Inclusion criteria: adult patient (≥18 years), emotionally unstable personality disorder (ie, ICD-10-CM^a^ diagnosis code: F60.3); referred to psychiatric treatment (ie, DBT^b^ psychotherapy); suicide attempt within the last 3 years Exclusion criteria: patients with schizophrenic spectrum disorders and bipolar disorder; substance abuse as primary problem (no desire to stop); intellectual problems (ie, IQ below 70)
Study type	Interventional Allocation: pragmatic, 2-arm, multicenter, open-label, evaluator-blind randomized controlled trial Active arm: self-registration with the Monsenso mobile app; control arm: self-report by pen-and-paper diary cards Primary purpose: intervention Phase III
Date of first enrollment	June 2017
Target sample size	80
Recruitment status	Active, not recruiting
Primary outcome(s)	Mean number of days required to learn a new DBT skill (time frame: 1 year)
Key secondary outcomes	Borderline severity (ZAN-BPD^c^), ability to regulate emotion (DERS^d^), compliance filling out diary cards

^a^ICD-10-CM: International Classification of Diseases, 10th Revision, Clinical Modification.

^b^DBT: dialectical behavior therapy.

^c^ZAN-BPD: Zanarini Rating Scale for Borderline Personality Disorder.

^d^DERS: Difficulties in Emotion Regulation Scale.

### Recruitment

Patients in the DBT treatment group for BDP and related problems will be recruited from five psychiatric outpatient units treating BDP (ie, Svendborg, Haderslev, Vejle, Silkeborg, and Glostrup) between August 2017 to December 2019. All sites have comprehensive DBT programs that have functioned as standard DBT programs for more than 7 years and include all five modes of DBT (ie, weekly consultation team; skills training groups; individual therapy, along with telephone coaching available outside of therapy sessions; and help structuring clients’ social and family environments if relevant [41]).

All patients will be informed orally and in writing about the research project. Consent for participation will be obtained not from the therapist of the patient but from an unbiased research assessor from the mDiary team. Patients were involved in designing the mDiary app.

Patients will be included in the study provided they fulfill all of the inclusion criteria and none of the exclusion criteria; they must also provide written informed consent, which will be collected by the therapists. Inclusion criteria are as follows: aged 18 years or older; a primary diagnosis of Emotionally Unstable Personality Disorder (F60.3 according to ICD-10-CM [International Classification of Diseases, 10th Revision, Clinical Modification] criteria) and diagnosed by a specialist in psychiatry (referred to as BPD here, the more common diagnostic label); referral for psychiatric treatment at the DBT treatment sites involved in the study; willingness to sign a commitment contract for DBT treatment; having had problems with self-harm or suicidal behavior within the last 3 years. Self-harm is defined as any form of self-inflicted tissue damage, excluding superficial scratching. Comorbid depression, anxiety, PTSD, antisocial traits or micro-psychotic episodes are not exclusion criteria as long as BPD is primary diagnosis. The above criteria will be screened through the Symptom Checklist-90-R (SCL-90-R) questionnaire subscores as well as targeted questions during the assessment interview. Patients with secondary substance use will be included if they agree to work on reducing their abuse.

The exclusion criteria are as follows: any diagnosis of schizophrenic spectrum disorders (any type of schizophrenic disorder or schizotypal personality disorder); any diagnosis involving bipolar disorder or a comorbid diagnosis of substance abuse disorder without a wish to change the associated behavior. The exclusion criteria will be checked in the electronic patient journal and through structured interviews during the intake procedure. Patients with intellectual problems comparable to an IQ below 70 will be excluded. Screening for intellectual disability will be done with the Danish Adult Reading Test (DART) [42]. Lack of a mobile phone or participation in other concomitant psychotherapy will also be reasons for exclusion.

### Statistical Analysis

The primary endpoint is the skills learning rate—the amount of time (in days) taken to learn a new skill (ie, progress from “started learning the skill” to “have learned the skill”). Patients assess and switch status using a button on the mobile phone app when they consider the skill is of use to them; at this time, the skill is considered learned, which will then be discussed and confirmed with the therapist during the preceding session.

The secondary endpoints are (a) BDP symptoms, including the ability to regulate emotion, will be evaluated with the interview-based ZAN-BPD; (b) percentage of completed diary questions, measured as a day with an entry in the mDiary app or in the paper-based diary; (c) quality of life measured by the EuroQoL five-dimension, five-level (EQ-5D-5 L) instrument; and (d) depression measured with the Patient Health Questionnaire (PHQ-9).

### Power

We are planning a study of 80 patients with equal allocation to each arm, an accrual interval of 360 days, and an additional follow-up of 360 days. The sample size of 80 patients is a realistic recruitment goal for the given timeframe. To our knowledge, no studies have previously investigated DBT skill acquisition time through the use of mobile phones. Based on pilot data and relevant noticeable clinical differences, it is estimated that the mean time to learn a DBT skill will be 1.5 month (44 days) for mobile phone registration versus 2 months (60 days) for the control condition. Using an alpha of .05 and a power of .90, we will need 437 events of learned skills in total to reject the null hypothesis. With an expected attrition rate during follow-up of 20%, each remaining patient (n=64 will on average need to generate 7 learned skills. A very cautious estimate would be that half of the patients (32 patients) will learn half of the skills (16 skills) during one year of therapy. This would generate 512 events, meaning that an inclusion of 80 patients will be able to generate sufficient power. A more realistic expectation is that 75% of the patients following protocol will learn 75% of the skills, which will generate 1188 events and will leave ample room to adjust for shared frailty. It is estimated that at least 15,000 episodes of a specific skill use will contribute to the survival analysis. The power analysis was performed using R statistical software and gsDesign. The power calculation is based on the log-rank test [43].

### Randomization

All BPD patients in DBT treatment group are offered participation in the study. All patients are followed from the beginning of their therapy. Randomization and initial assessments are done by an independent research assistant. Initial assessment is completed by a blinded assessor since it is done before randomization. Stratification is done by site as well as by severity, aiming for equal distribution of severe and less severe cases within each site. Severity is assessed by the general severity score from the SCL-90-R questionnaire with a Global Symptom Index cut-off score of ≥1.75. Blocks of four are randomized with a ratio of 1:1. The allocation sequence is generated by a random number computer algorithm transferred to two stacks of sealed envelopes (severe and less severe) for each site that is opened by the participant immediately after the first assessment. The study needs to be open label since it is not possible to withhold the treatment condition (paper or mobile phone), but the analysis will be performed blinded.

### Training of Therapists

The therapists at the five sites will be trained to use the app during two meetings, each one hour in length, and agree to refer patients to the research project. The sites have an estimated intake of 20 patients per site per year. In case of reduced enrollment, the recruitment period will be prolonged. Each site has 4-7 active therapists, typically 1 psychiatrist, 1-2 psychologists, and 1-2 nurses specializing in DBT. Referrals to the clinics are from either a primary care physician or from the inpatient section of the hospital ward dealing with acute psychiatry. All therapists are trained in standard DBT.

### Intervention

All patients in the mDiary trial will receive standard DBT treatment. The difference between the two conditions in the trial is whether self-monitoring is done with the pen-and-paper method or the Monsenso system. There is a higher degree of interactivity and available information on the Monsenso system, as it is possible to access submenus on a mobile phone app to learn more about what patients report. Descriptions of the skills to be learned include more details, as well as a larger psychoeducative element. Skills explanations are supported by sound files.

### Content of the Monsenso System

**Figure 2:**
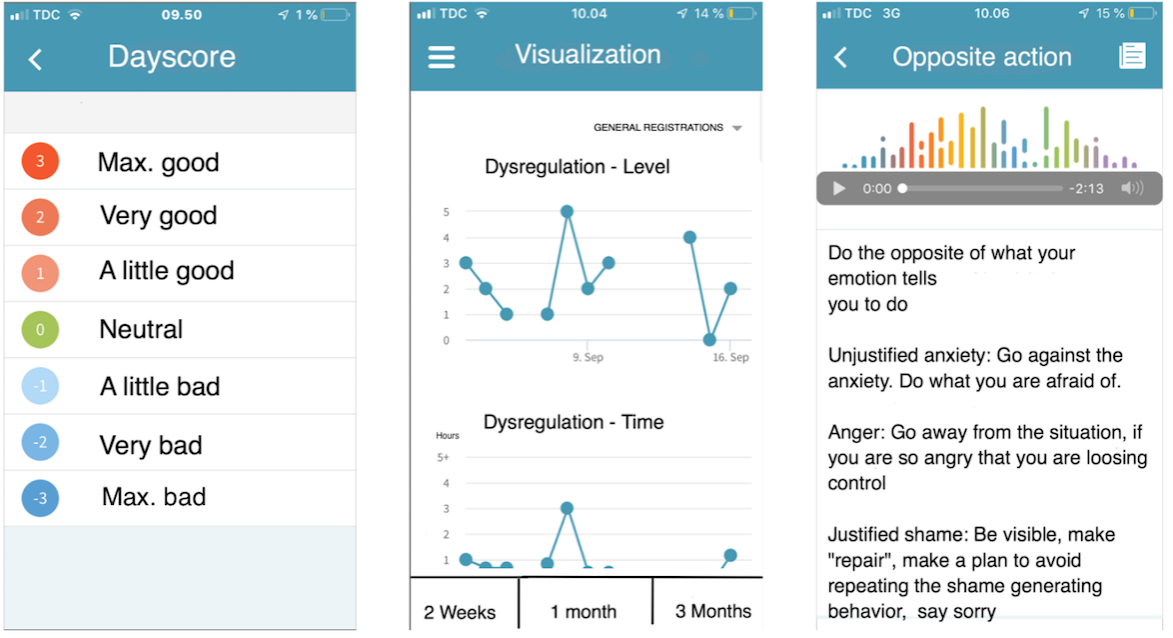
Screenshots of the Monsenso system.

Figure 2 displays three mobile phone screenshots of the Monsenso system. The screen to the left is an example of registering one of the required variables (ie, day score). Under normal circumstances, this is one of 10 variables participants would register daily. This score is a general rating of the day in terms of good or bad. The middle screenshot is a visualization of variables entered that can be seen after registering all variables of the day. It is possible to scroll down to see more registered variables. The right-hand screenshot explains one of the DBT skills (ie, opposite action). This is delivered in both text and podcast format.

**Figure 3:**
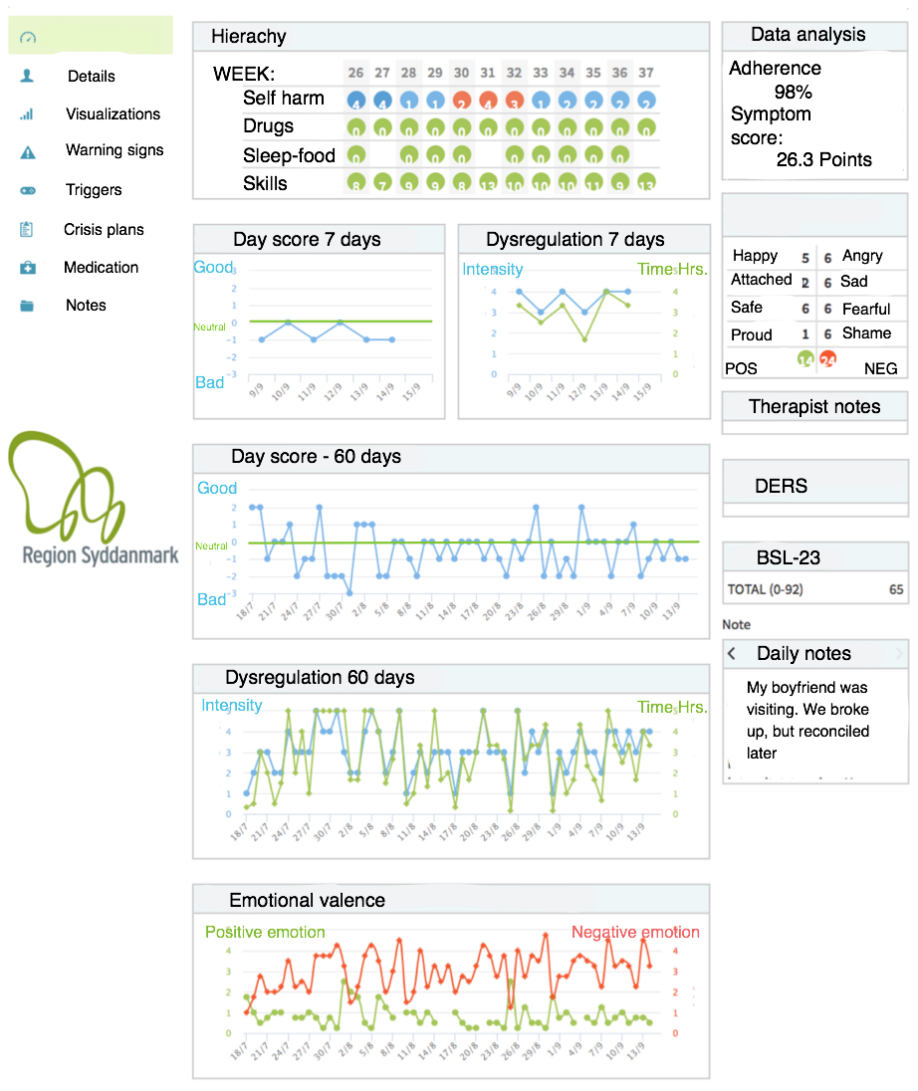
Overview screen for the therapy session.

An overview screen for the therapy session is provided in Figure 3. Summed scores for the treatment and the DBT hierarchy by week number are displayed. Red dots signal self-harm or suicidal behavior. Aggregated scores for drug use, vulnerability factors, and skill use are shown as well. The next row presents visualizations of dysregulation and day scores by day. Taken together, this provides a quick overview of the week. The next two rows display the day score and dysregulation for the past 60 days, providing a longer overview of development. A separate, dedicated screen can be accessed for even longer time spans (ie, two years or more). The bottom row shows aggregated scores for positive and negative emotions in the past 60 days. The top right side of the screenshot provides the compliance percentage (adherence) for completed diary cards in the past 60 days, as well as a total aggregated score of severity (symptom score). Below that, the aggregated scores of positive and negative emotions are broken down based on the number of days different emotions were registered. Below that, there is room for therapist notes and the Borderline Symptom List (BSL-23) and the Difficulties of Emotion Regulation (DERS) summed scores. In the bottom right, patient notes from their diary dating to the last 7 days is accessible. If a score from the previous week is particularly interesting, there will be a quick way to gain more information through a link to the comments for that specific day. All patient diary notes are accessible via a separate, dedicated screen.

The left-hand row is a menu for accessing other overview screens like the long-term overview of variables, program notifications like triggers and warning signs, a dedicated screen for diary notes, medication, and construction of action plans.

### Data Collection and Management

Daily registrations for the diary are collected through the Monsenso system or on a piece of paper that closely resembles the standard DBT paper diary sheet. Collected variables are listed in Table 2. The column “Always” shows which variables are always collected through the mobile phone on a daily basis. The column “When relevant” shows variables that can be switched off when they are no longer relevant. Patients for whom emotions are too painful to register at the start of the treatment can switch off the variable in the beginning. “Self-harm” can be switched off when treated to a completely extinct level; patients are instructed to switch this registration on, if they are to relapse.

Questionnaires for overall assessment of all participants are collected through the REDCap (Research Electronic Data Capture) system. All data collected are monitored through the Odense Patient Data Exploratory Network (OPEN) using a logged secure database run by the Region of Southern Denmark. Paper diaries will be entered by double data entry into the OPEN database, securing data quality and data safety. The OPEN team ensures that the data are stored according to the European Union’s General Data Protection Regulation (GDPR) standards and CONSORT guidelines. The research data will only be accessible to the research team. An anonymized version will be stored at the Danish National Archives (Rigsarkivet) in order to revisit, extend, and validate conclusions from the RCT.

**Table 2:** Outcomes collected daily from the mobile phone–based or paper diary.

Covariate	Always	When relevant
Skill use (3 factors: unknown, started learning it, have learnt it)	✔	
Dysregulation, intensity	✔	
Dysregulation (duration in hours/day)	✔	
Numbness	✔	
Day score (–3 to +3)	✔	
Qualitative short description of the day	✔	
Suicide thoughts and actions (0-5)		✔
Self-harm thoughts and actions		✔
Basic emotions (anger, joy, shame, pride, love, sad, anxious, safe; 0 to +3)		✔
Eating (too little, balanced, or too much; –3 to +3)		✔
Drugs		✔

Deviations from participating in the intervention will not be addressed in order to mirror normal participation in outpatient treatment as closely as possible. Patients who decline to participate in the study will be assessed by their therapist, who will fill out a form stating reasons for not wanting to participate.

A dedicated data safety monitoring board to protect participants from aversive consequences of the intervention will not be necessary since the study is open label. Patients are followed on a day-to-day basis by psychotherapists and patient safety will be monitored weekly by therapists, which makes an unblinded double check redundant. Adherence to filling out forms on paper and through the mobile phone app (days with entries) will be part of the secondary outcome measurements and will not be sought or influenced during data collection.

### Outcomes Collected

Patients are asked to complete a set of questionnaires during the study period (Table 3). These are delivered by mobile phone, thereby posing a smaller burden than standard questionnaires. Questionnaires are sent out automatically to the patients’ mobile phone and does not require the patient to meet with an assessor at a specified time and place. It can be filled out when convenient during the day.

**Table 3:** Questionnaires used in the study.

Questionnaire	Questions, n	Frequency	Endpoint	Reference
Danish Adult Reading Test (DART)	40	Only pre	DART measures the number of correctly pronounced words when the patient reads out loud	Hjorthøj et al [42]
Zanarini Rating Scale for Borderline Personality Disorder (ZAN-BPD)	9	Pre, post	ZAN-BPD measures clinician rated BPD^a^ severity	Zanarini et al [44]
Suicide Behaviors Questionnaire-Revised (SBQ-R)	4	Pre, post	SBQ-R measures suicide behavior	Osman et al [45]
Posttraumatic Stress Disorder-8 items (PTSD-8)	8	Pre, post	PTSD-8 consists of 8 questions from the Harvard Trauma Questionnaire for screening PTSD^b^ severity	Hansen et al [46]
Symptom Checklist-90-R (SCL-90-R)	90	Pre, post	SCL-90-R measures self-perceived psychiatric symptom load	Brophy et al [47]
EuroQoL five-dimensions, five-levels (EQ-5D-5L)	5	Pre, post	EQ-5D-5L measures quality of life for health economic evaluation	Janssen et al [48]
Patient Health Questionnaire (PHQ-9)	9	Pre, post	PHQ-9 measures suicidality and depressive symptoms for health economic evaluation	Kroenke et al [49]
Treatment Inventory Cost in Psychiatric patients (TIC-P)	11	Pre-post	TIC-P measures treatment costs and loss of productivity for health economic evaluation	Bouwmans et al [50]
Self-Harm Inventory (SHI)	12	Pre, post	SHI measures lifetime self-harm type and count	Sansone et al [51]
Borderline Symptom List (BSL-23)	23	Monthly	BSL-23 measures BPD core symptoms	Wolf et al [52]
Difficulties in Emotion Regulation Scale (DERS)	36	Monthly	DERS measures ability to regulate emotion	Weiss et al [53]
Positive and Negative Affect Schedule short form (PANAS SF)	10	Weekly	PANAS SF comprises 10 items and measures positive and negative affect	Watson et al [54]

^a^BPD: borderline personality disorder.

### Statistical Analysis

All analyses will be conducted according to the intention-to-treat principle. Comparison between the active and control conditions on the primary endpoint will be performed by means of survival curves, as time-to-event models analyzed by Cox proportional hazards regression models with multiple events per patient. Significance tests are based on a frailty mixed effects approach [55]. Since missing data is not expected to be missing randomly, data will be modelled with multiple imputations, if missing data is to be included.

The level of dependence or heterogeneity among patterns of skill acquisition speed within the same site will also be explored by the intracluster correlation coefficient (ICC) [56]. Apart from intervention type (mobile phone or paper), treatment site, intellectual ability (DART score), level of BPD (ZAN-BPD), and level of PTSD (PTSD-8) are used as interaction terms in the Cox proportional hazards regression models.

A secondary analysis of differences in compliance in filling out daily registrations between mobile phone and paper groups will use Wilcoxon rank sum to test the hypothesis of improved compliance in the active arm of the intervention. The problem of backfilling paper diary entries [57] cannot be easily controlled in the pen-and-paper version, but in the mobile phone condition, it will only be possible to back fill 2 days’ worth of entries, so clusters of 3-day fill-outs will signal backfilling. This bias in the study will be addressed in the interpretation of the results.

On an explorative level, time series data from daily data collection will be used to predict a binary classification of treatment in responders and nonresponders. In this analysis, the classification of responders and nonresponders will be based on the definitions provided by Jacobson et al [58] and and Schmitgen et al [59].

### Adverse Effects

Due to the nature of BPD, self-harm and suicidal behavior might appear during psychotherapy, but the DBT treatment is aimed at dealing with this type of behavior. It is safe to assume that monitoring symptoms and skills acquisition on a mobile phone instead of paper forms will not subject patients to additional risk. During the DBT treatment, patients are coached in dealing with suicidal and self-harm behaviors. Furthermore, a therapist or an acute team is available by telephone 24/7 for patients. Even if suicidal ideation should emerge, there is a very quick path to rapid crisis intervention. If unforeseen adverse effects should emerge due to the study, these problems will be quickly presented to the mDiary RCT advisory board for evaluation, and matters will be responded to immediately.

### Patient Involvement Statement

Patients were involved in the pilot and development phases, by engagement in the codevelopment of the mobile app. The research question reflects a reoccurring wish for mobile phone registration from patients in DBT treatment, making self-monitoring less like homework from school and more technologically up to date.

### Data Availability

The data sets generated and analyzed during the current study are not publicly available due to the highly sensitive nature of the content. Psychiatric diagnosis, suicidality, self-harm, and alcohol and drug use are disclosed in the diary registrations. It is not possible to do this kind of research without promising the research subjects the highest level of confidentiality, that is, complete data anonymization. Relatively few study subjects were treated at each site, thus making it possible to infer site location by looking at the number of subjects treated at the sites. Very few men participated in the study, so the identity of single individuals may be inferred by looking at the sex and location data. However, the data can be made available in a nonidentifiable form from the corresponding author on reasonable request.

### Ethics and Dissemination

The study was approved by the regional Committees on Health Research Ethics for Southern Denmark (Journal number: S-20160085) and the Danish data registry (Journal number: 2008-58-0035). The trial was registered on ClinicalTrials.gov (NCT03191565) on June 19, 2017 and is conducted in agreement with the Declaration of Helsinki. Informed consent material is available in Danish with the approved protocol. If a patient sustains any trial-related harm, they are covered by Danish law. The Committees on Health Research Ethics select and audit a number of studies on an annual basis. Independent investigators and sponsors conduct the audit process. The primary investigator has unrestricted access to the full data set. There are no contractual limitations regarding access of the full data set for relevant investigators. There are no restrictions regarding the dissemination of results. Primary authorship will be held by the primary investigator when reporting the results of the study.

### Funding

This study is part of a larger project (the ENTER [Programme for E-meNTal hEalth Research] project) at the Centre for Telepsychiatry in the Region of Southern Denmark. In January 2016, €2.7 million for the ENTER program was obtained from the Danish Innovation Fund Denmark [60] (grant number 5159-00002B). The funding body does not have any ownership or authority over the data collected.

## Results

All results will be written up for publication and submitted to international peer-reviewed journals. Positive as well as negative or inconclusive findings will be published. Results will be reported according to the updated guidelines for reporting parallel group randomized trials—the CONSORT 2010 Statement—and the guidelines for the inclusion of patient-reported outcomes in clinical trial protocols—the SPIRIT (Standard Protocol Items: Recommendations for Interventional Trials) extension. Significant protocol amendments will be reported back to the ethics committee, the Danish data registry, and ClinicalTrials.gov, and will be reported in the primary results paper. Authorship will be granted to all participating authors, according to the current principles stated by the International Committee of Medical Journal Editors.

## Discussion

The present effectiveness trial focuses on individuals with BPD, who have a high level of service utilization, much subjective suffering, a high suicide rate, and high-cost treatments [61].

The results of the RCT will be a first attempt at giving an internet-based, mobile phone solution an evidence base to operate from. This study adds to the literature by providing and assessing best practice in terms of self-monitoring during BPD psychotherapy. The exploration of trajectories of improvement or deterioration may point to new uses of diary registration during psychiatric treatment. A promising aspect of the approach is the collection of diary data through the patients’ own mobile phones. Data is entered directly into a database without further action from the clinician. Thus, data is stored very conveniently on a server making both short- and long-term review accessible, as well as facilitating bench marking and detailed record keeping. As databases will naturally grow with the passing of time and data collection, this data collection method will be increasingly amenable to machine learning. In future psychotherapy treatments, this mode of self-monitoring could inform us of predictors and relevant classification from start to follow-up.

## Abbreviations

BPD: borderline personality disorder
BSL-23: Borderline Symptom List
CONSORT: Consolidated Standards of Reporting Trials
DART: Danish Adult Reading Test
DBT: dialectical behavior therapy
DERS: Difficulties in Emotion Regulation Scale
ENTER: Programme for E-meNTal hEalth Research
EQ-5D-5L: EuroQoL five-dimension, five-level
GDPR: General Data Protection Regulation
ICC: intracluster correlation coefficient
ICD-10-CM: International Classification of Diseases, 10th Revision, Clinical Modification
OPEN: Odense Patient Data Exploratory Network
PANAS SF: Positive and Negative Affect Schedule short form
PHQ-9: Patient Health Questionnaire
PTSD-8: Posttraumatic Stress Disorder-8 items
SBQ-R: Suicide Behaviors Questionnaire-Revised
SCL-90-R: Symptom Checklist-90-R
SHI: Self-Harm Inventory
SPIRIT: Standard Protocol Items: Recommendations for Interventional Trials
TIC-P: Treatment Inventory Cost in Psychiatric patients
ZAN-BPD: Zanarini Rating Scale for Borderline Personality Disorder
